# Serum biomarkers in patients with ossification of the posterior longitudinal ligament (OPLL): Inflammation in OPLL

**DOI:** 10.1371/journal.pone.0174881

**Published:** 2017-05-03

**Authors:** Yoshiharu Kawaguchi, Masato Nakano, Taketoshi Yasuda, Shoji Seki, Kayo Suzuki, Yasuhito Yahara, Hiroto Makino, Isao Kitajima, Tomoatsu Kimura

**Affiliations:** 1 Department of Orthopaedic Surgery, University of Toyama, Toyama, Japan; 2 Department of Clinical Laboratory Medicine, University of Toyama, Toyama, Japan; Mayo Clinic Minnesota, UNITED STATES

## Abstract

**Backgroud:**

Ossification of the posterior longitudinal ligament (OPLL) is characterized by replacement of ligamentous tissue by ectopic new bone formation. OPLL causes narrowing of the spinal canal, resulting in neurological impairment. However, the pathogenesis of OPLL has not been fully elucidated. We investigated whether inflammation occurs in OPLL or not using high-sensitivity CRP (hs-CRP) in a case-control study.

**Methods and findings:**

This study included 103 patients with OPLL in the patient group and 95 age- and sex-matched volunteers with degenerative spinal disease in the control group. Of the 103 OPLL patients, 88 patients who were available for more than 2 years follow-up were checked for OPLL progression. A blood sample was obtained and Hs-CRP, and other routine data, including total protein (TP), albumin (ALB), lactate dehydrogenase (LDH), alkaline phosphatase (ALP), glucose (Glu), calcium (Ca), inorganic phosphate (Pi), white blood cell count (WBC), hemoglobin (Hb) and platelet (PLT), were analyzed. The data were compared between the patients with OPLL and the controls. The severity of the ossified lesions in the whole spine were evaluated by the ossification index (OS index) in patients with OPLL. The data were also compared between the patients with OPLL progression (the progression group) and the patients without OPLL progression (the non-progression group). In the results, the mean hs-CRP in the OPLL group was higher than that in the controls. The Pi in the OPLL group was lower than that in the control group. A negative correlation was found between the Pi and the OS index. The mean hs-CRP in the progression group was higher than that in the non-progression group. There was a positive correlation between the average length of the OPLL progression per year and the hs-CRP.

**Conclusions:**

The results may suggest the occurrence of local inflammation in OPLL and the inflammation might cause OPLL progression. These facts are important for understanding the pathology of OPLL.

## Introduction

**Fig 1:**
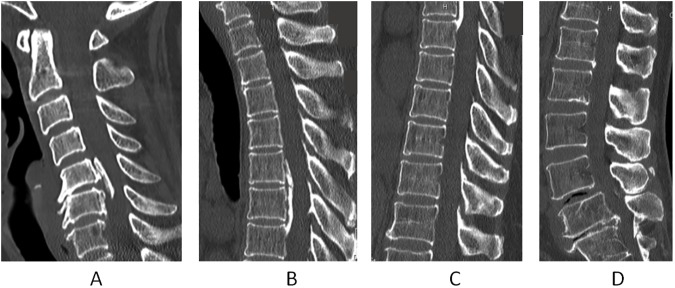
CT images of a patient with ossification of the posterior longitudinal ligament (OPLL). This 60-year-old female has OPLL at C5, 5–6, 6 (A), T5, 5–6, 6 6–7,7 (B, C) L1-2 and L5-S1 (D). Her OS index is 9.

Ossification of the posterior longitudinal ligament (OPLL) is characterized by ectopic new bone formation which serves as the replacement for ligamentous tissue (Fig 1) [1,2]. OPLL often causes narrowing of the spinal canal and it has been recognized as one of the causes of neurological impairment, such as cervical myelopathy and/or radiculopathy [1,2]. Many OPLL cases have been reported in Japan. This disease was thought to occur more frequently in Asians, however, OPLL is now known to also be observed in Caucasians [3]. Although the pathogenesis of OPLL has not been fully elucidated, a genetic background for systemic ossification could be involved in OPLL [4]. To date, 6 candidate genes have been identified in the whole genome association study [4]. A generalized hyperostotic tendency is associated with the pathogenesis of OPLL [5–7]. Resnick et al [8] reported that OPLL is considered to be a partial phenotype of diffuse idiopathic skeletal hyperostosis (DISH), which is characterized by ossification of not only the spinal ligaments, such as the anterior longitudinal ligament, ligamentum flavum, and interspinous ligament, but also the systemic periarticular ligaments. It has been reported that bone morphogenetic protein-2 (BMP-2) and transforming growth factor-β (TGF-β) are related to the pathogenesis of OPLL [9]. A recent research article suggested that OPLL is mediated by osterix via a mechanism involving the Wnt/β-catenin signaling pathway [10]. Based on these results, a systemic increase of bone formation activity in OPLL patients has been suggested. We have shown that the serum concentrations of intact osteocalcin, osteocalcin and carboxyterminal propeptide of human type I procollagen reflect the activity of general ectopic bone formation in OPLL patients [11]. It has been reported that several biomarkers, such as leptin and insulin, are related to OPLL [12,13]. However, we do not know whether or not inflammation occurs in OPLL. A previous study demonstrated that the CRP level is increased in patients with heterotopic ossification after total hip replacement [14]. To date, no study has discussed inflammation in patients with OPLL. As the ossified lesions in OPLL are very small, they may not influence the level of CRP. Studies employing the ultrasensitive latex-enhanced immunoassay (Latex) [15] for high-sensitivity CRP (hs-CRP) concentration analysis have reported that hs-CRP is useful in the prediction of myocardial infarction and stroke [16–19]. Thus, it is hypothesized that hs-CRP might be useful to detect slight inflammation at ectopic bone formation sites in the spinal ligaments. The present study was prospectively designed to determine whether or not the serum CRP concentration and other biomarkers are altered in patients with OPLL in comparison with the age- and sex-matched controls with degenerative spinal disease. Further, for cases showing a difference in the serum CRP concentration and/or other biomarkers, we performed analyses to determine the factors related to the difference.

## Materials and methods

The patient group comprised 103 (63 men and 40 women) patients with cervical OPLL with an average age of 68.1 ± 10.0 years (range, 41–85 years). For the control group, 95 (53 men and 42 women, average age 69.9 ± 10.1 years, range 44–89 years) age- and sex-matched volunteers with degenerative spinal disease were included. All patients with OPLL and controls were recruited from 2012 to 2014 in the Department of Orthopaedic Surgery at Toyama University Hospital, Japan. Informed written consent was obtained from all participants indicating their willingness to attend the study. The study protocol was approved by the Ethics Committee of the University of Toyama. The diagnosis of OPLL was based on radiological findings, including radiographs and computed tomography (CT) scans of the cervical, thoracic, and lumbar spine. Ankylosing spondylitis and metabolic diseases associated with OPLL, such as hypophosphatemic rickets/osteomalacia and hyperparathyroidism, were excluded based on radiographic and biochemical examinations. A total of 88 patients with OPLL (53 men and 35 women: average age 68.3 ± 10.4 years, range 41–85 years) were available for a follow-up of more than 2 years with radiological examinations. The average follow-up length was 5.2 ± 2.1 years (range, 2–10 years). Plain radiographs were used in 34 patients and CT images were used in 54 patients for the evaluation of the ossified lesions of OPLL and to determine OPLL progression. OPLL lengthening of more than 2 mm during the follow-up was judged to be “OPLL progression”. The controls were age-matched patients with a diagnosis of cervical spondylosis, lumbar degenerative disease and/or spinal disc disease. Of the 95 controls, 19 had cervical spondylosis, 68 had lumbar degenerative disease, 4 had cervical disc herniation and 4 had lumbar disc herniation. The diseases were confirmed by image studies, including plain radiographs, CT and MRI. None of the controls had spinal canal ossifications, as confirmed by CT. Any OPLL patient or control subject with inflammatory diseases (such as collagen diseases and rheumatoid arthritis), infections, trauma, myocardial infarction, cerebral infarction or malignant tumors was strictly excluded. No patient or control subject was taking non-steroidal anti-inflammatory drugs and/or steroids. The demographic data of the OPLL group and the control group are shown in Table 1.

**Table 1:** Demographic data of the patients with OPLL (case) and the control.

	OPLL	control	p
gender (M/F)	63/40	53/42	0.44
age (year)	68.1±10.0	69.9±10.1	0.21
height (cm)	161.2±8.8	159.0±9.4	0.12
weight (Kg)	63.1±13.9	60.0±11.7	0.08
BMI	24.1±4.3	23.6±3.5	0.37

BMI: body mass index.

A blood sample was obtained from all participants in the morning of the hospital visit. The hs-CRP was analyzed using an ultrasensitive latex-enhanced immunoassay (L-Latex CRP II) employing the BN ProSpec nephelometer (Dade Behring, Newark, DE) [15]. This immunonephelometric assay is a high-sensitivity assay capable of measuring hs-CRP at a concentration of 0.00095 mg/dl [20]. Other routine clinical lab tests, including total protein (TP), albumin (ALB), lactate dehydrogenase (LDH), alkaline phosphatase (ALP), glucose (Glu), calcium (Ca), inorganic phosphate (Pi), erythrocyte sedimentation rate (ESR) at 1 hour and 2 hours, white blood cell count (WBC), hemoglobin (Hb) and platelet count (PLT), were also assessed. The data were compared between the patients with OPLL (the OPLL group) and the controls. Also data were compared between the patients with OPLL progression (the progression group) and the patients without OPLL progression (the non-progression group).

In the patients with OPLL, the severity of the ossified lesions in the whole spine was evaluated using our previously published ossification index (the OS index) [21]. This index is determined by the sum of the levels of vertebral bodies and intervertebral discs where OPLL is present. When the ossification area extends from the vertebral body level to the intervertebral disc level, ossified lesions of each vertebral body level and intervertebral disc level are counted. Theoretically, the maximum OS index is 14 in the cervical spine. The OS index in the thoracic spine ranges from 0 to 24 and that in the lumbar spine is from 0 to 11.

## Statistical analysis

Chi-square test was used for the difference in gender between the OPLL group and the control group. The same statistical method was used for the difference between the OPLL progression group and the non-progression group. Age, height, weight and body mass index, as well as the serum concentrations, were presented as mean ± standard deviation. Differences between groups were analyzed for statistical significance using Student’s t test (unpaired). Simple linear regression and Pearson’s correlation were used to analyze the correlation between the OS index and several biomarkers and also between the progression length of OPLL per year and hs-CRP in serum. StateMate V software (ATMS, Tokyo, Japan) was used for the analysis and p<0.05 was considered as statistically significant.

## Results

Table 2 shows the comparison of the serum biomarkers between the OPLL group and the controls. The mean serum hs-CRP concentration was 0.122 ± 0.141 mg/dL in the OPLL group and 0.086 ± 0.114 mg/dL in the controls, yielding a statistical difference between the two groups (p = 0.047). ESR-1h and ESR-2h in the OPLL group were higher than those in the control group (p = 0.003, p = 0.002, respectively). Although the serum concentration of Ca did not differ in the two groups, the concentration of Pi in the OPLL group was lower than that in the control group (p = 0.02).

**Table 2:** Comparison of the biomarkers between the patients with OPLL (case) and the controls.

		OPLL group	control group	p
TP	(g/dL)	7.13 ± 0.68	7.03 ± 0.53	0.25
ALB	(g/dL)	4.19 ± 0.36	4.12 ± 0.33	0.15
LDH	(U/L)	200 ± 35.9	201 ± 35.6	0.84
ALP	(U/L)	248 ± 70.9	233 ± 69.4	0.12
Glu	(mg/dL)	122 ± 44.2	118 ± 37.0	0.5
Ca	(mg/dL)	9.11 ± 0.35	9.20 ± 0.44	0.11
Pi	(mg/dL)	3.19 ± 0.55	3.36 ± 0.47	0.02
hs-CRP	(mg/dL)	0.122 ± 0.141	0.086 ± 0.114	0.047
ESR-1h	(mm)	16.7 ± 15.4	10.9 ± 7.5	0.003
ESR-2h	(mm)	34.6 ± 24.8	25.2 ± 15.4	0.002
WBC	(X100/μL)	67.3 ± 55.3	61.3 ± 15.5	0.29
Hb	(g/dL)	14.4 ± 4.6	13.9 ± 3.3	0.38
PLT	(X10000/μL)	21.6 ± 5.7	21.5 ± 5.9	0.9

TP: total protein, ALB: albumin, LDH: lactate dehydrogenase, ALP: alkaline phosphatase, Glu: glucose, Ca: calcium, Pi: inorganic phosphate, ESR: erythrocyte sedimentation rate at 1 hour and 2 hours, WBC: white blood cell count, Hb: hemoglobin, PLT: platelet count, OPLL: ossification of the posterior longitudinal ligament.

**Fig 2:**
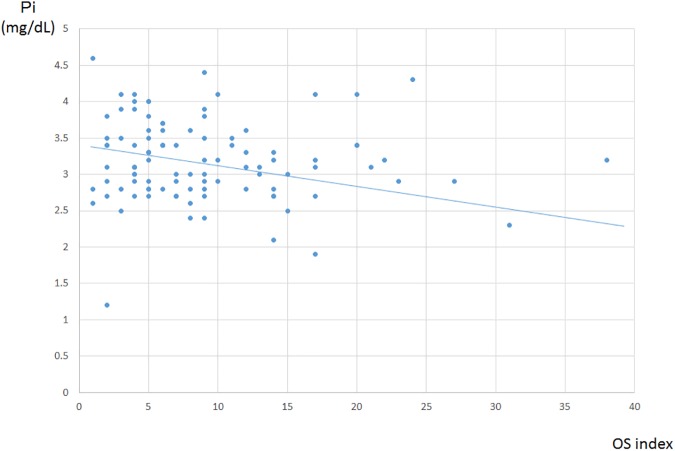
The relationship between the serum concentration of Pi and the OS index in the OPLL group. A negative correlation was found. (p<0.001, r = -0.51).

The average OS index of the total spine was 9.2 ± 6.8 and it ranged from 1 to 38. There was no relationship between hs-CRP and the OS index. However, a negative correlation was found between the serum concentration of Pi and the OS index. The patients with a high OS index showed a lower concentration of Pi (Fig 2, p<0.001, r = -0.51).

The demographic data of the progression group and the non-progression group are shown in Table 3. There was no difference in the follow-up length between the two groups. The age in the progression group was younger than in the non-progression group. The male patients in the progression group were significantly taller and heavier than those in the non-progression group.

**Table 3:** Demographic data of the progression group and the non-progression group.

	progression group	non-progression group	p
gender (M/F)	`23/9	30/26	0.09
age (year)	64.4±10.3	70.5±9.8	0.006
male height (cm)	167.8±5.2	164.7±6.2	0.048
female height (cm)	153.2±9.5	153.4±6.9	0.95
male weight (Kg)	71.5±13.9	64.3±11.0	0.04
female weight (Kg)	61.6±15.3	56.0±9.6	0.33
male BMI	25.3±4.0	23.7±3.4	0.12
female BMI	26.0±5.0	23.7±3.3	0.23
follow-up length (year)	5.6±2.0	4.9±2.2	0.13

BMI: body mass index.

Table 4 shows the comparison of the serum biomarkers between the progression group and the non-progression group. The mean serum hs-CRP concentration was 0.18 ± 0.15 mg/dL in the progression group and 0.099 ± 0.14 mg/dL in the non-progression group, yielding a statistical difference between the two groups (p = 0.0013). There were no differences among the other biomarkers in the two groups.

**Table 4:** Comparison of the biomarkers betweeen the progression group and the non-progression group.

		progression group	non-progression group	p
TP	(g/dL)	7.23 ± 0.84	7.13 ± 0.54	0.54
ALB	(g/dL)	4.23 ± 0.32	4.21 ± 0.34	0.78
LDH	(U/L)	196± 41.5	199 ± 31.4	0.72
ALP	(U/L)	248 ± 71.4	251 ± 69.8	0.85
Glu	(mg/dL)	120 ± 44.3	120 ± 42.9	1
Ca	(mg/dL)	9.12 ± 0.4	9.09 ± 0.32	0.72
Pi	(mg/dL)	3.05 ± 0.56	3.19 ± 0.52	0.25
hs-CRP	(mg/dL)	0.18 ± 0.15	0.099 ± 0.14	0.0013
WBC	(X100/μL)	62.6 ± 15.3	59.6 ± 16.4	0.39
Hb	(g/dL)	14.1 ± 1.93	13.6 ± 1.77	0.23
PLT	(X10000/μL)	21.4 ± 6.2	22.6 ± 6.8	0.4

TP: total protein, ALB: albumin, LDH: lactate dehydrogenase, ALP: alkaline phosphatase, Glu: glucose, Ca: calcium, Pi: inorganic phosphate, WBC: white blood cell count, Hb: hemoglobin and PLT: platelet count.

**Fig 3:**
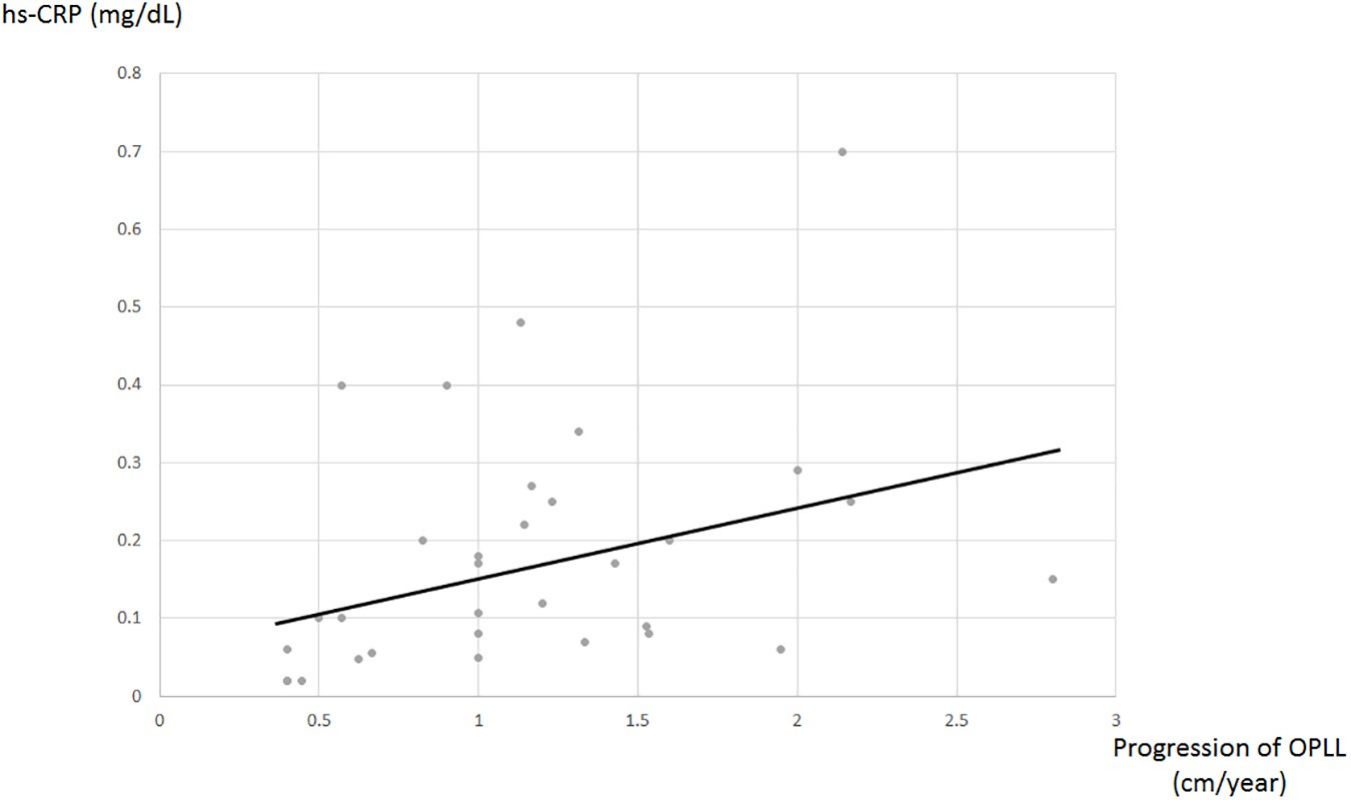
The relationship between the average length of OPLL progression per year and the serum concentration of hs-CRP in the OPLL group. A weak positive correlation was found. The data showed statistical significance (p = 0.045, r = -0.36).

The average length of the OPLL progression during follow-up was 6.4 ± 4.2 mm, ranging from 2 to 21 mm, and that per year was 1.2 ± 0.6 mm. There was a positive correlation between the average length of the OPLL progression per year and hs-CRP in serum, although the correlation was very weak (Fig 3, p = 0.045, r = -0.36).

## Discussion

The current study revealed two important factors regarding the pathogenesis of OPLL: one being inflammation and the other, calcium phosphate metabolism. The serum concentration of hs-CRP in the OPLL group was higher compared to the control group and ESR was also significantly higher in the OPLL group. Further, this study showed that serum hs-CRP in the OPLL progression group was significantly higher than that in the non-progression group. These results suggested that local inflammation is associated with the pathogenesis of OPLL. CRP is one of the most useful acute phase markers to detect inflammation after tissue injury. Pro-inflammatory cytokines, such as interleukin 6 (IL-6), interleukin 1β and tumor necrosis factor alpha (TNF-α) are responsible for the induction of CRP synthesis in the liver [22]. Although the increase in CRP in the patients with OPLL was very small, on the order of 10^−2^ mg/dL, it was significant. The ESR increase might also be related to inflammation. There was no difference in the concentrations of ALB and Hb between the OPLL and non-OPLL groups. ESR is affected by ALB and Hb. Thus, the elevation of both hs-CRP and ESR might imply the presence of inflammation in OPLL. OPLL shows ectopic bone formation in the spinal ligaments and ossification progression is frequently observed [23–25]. Previous studies have demonstrated that the CRP level is increased in patients with heterotopic ossification following total hip replacement [14] and in those with heterotopic ossification after traumatic spinal cord injury [26,27]. Although we have not encountered any surgical specimens which show local inflammation at the ectopic bony lesion of OPLL, there is some evidence which suggests local inflammation at the lesion site. The endochondral ossification process is consistently observed in OPLL [28]. BMP-2 and TGF-β are present in the ossified matrix and in chondrocytes of adjacent cartilaginous areas of ossification of the posterior longitudinal ligament [29]. BMP-2 and BMP-7 induce local inflammation, which has been shown in the pathology [30,31]. Thus, these facts suggest that cytokines, such as BMP-2, induce local inflammation around the ectopic bony lesion of OPLL. Based on these results, ectopic bone formation in patients with OPLL might be caused by the inflammation in the spinal ligament.

The ossified lesions of OPLL often show progression in the follow-up after cervical laminoplasty. Our previous study demonstrated that more than 70% of the patients with cervical OPLL had OPLL progression more than 10 years after cervical laminoplasty [23]. It has been reported that an increase in the area of ossified lesions affects the surgical results after laminoplasty [23,32]. Fargen et al reviewed the progression of OPLL and offered insight into potential future surgical strategies for OPLL treatment [33]. We have previously evaluated the speed of OPLL progression and showed that the inclination of simple regression, which means the average increase per year in younger patients in their fourth decade, was 2.38 ± 1.07 mm /year, whereas the data in older patients in their fifth decade was 0.61 ± 0.41 mm /year [24]. The speed was higher in the younger patients compared to that in the older patients. Based on that result, we considered that since OPLL is often found in patients in their 40s and 50s, it might be that there is a rapid increase in the fourth decade and then the progression gradually decreases in the fifth or sixth decades [24]. The present findings are in accordance with the previous study. In the hereditary spinal hyperostotic mouse (ttw mouse), which is the model mouse of OPLL [34], Uchida et al found that the enlargement of the nucleus pulposus followed by herniation, disruption and regenerative proliferation of annulus fibrosus cartilage tissues is involved in the initiation of ossification of the posterior longitudinal ligament [35]. They also found that the cells of the protruded hyperplastic annulus fibrosus invade the longitudinal ligament and induce neovascularization and metaplasia of primitive mesenchymal cells to osteoblasts in the spinal ligaments. The current study revealed that there was a weak positive correlation between the average length of the OPLL progression per year and the serum concentration of hs-CRP. This data imply that a more rapid progress of OPLL reflects a higher increase in hs-CRP. This also might suggest that the severity of local inflammation is related to the speed of OPLL progression. These active local pathology findings might reflect the inflammation which was detected by hs-CRP.

In the current study, the serum concentration of Pi in the OPLL group was lower than that in the controls, which is in accordance with the previous study by Takuwa et al [36], who reported that patients with paravertebral ligament ossification showed a lower serum Pi concentration than controls, although the study size was very small. In their study, the number of the patients and controls was 28 and 11, respectively. Pi, as well as Ca, plays an important role in skeletal development and bone mineral metabolism [37]. However, the fact that there was no difference in the serum concentration of Ca between the OPLL group and the controls suggests that Pi might be a key factor in OPLL. In fact, a recent case report revealed that 34-year-old female with vitamin D resistant rickets had extensive ossification of the paraspinal ligaments. The serum concentration of her Pi was low whereas that of Ca was normal [38,39].

The ttw mouse develops an ectopic bone formation in the spinal ligament which results in severe myelopathy [34,35]. It has been identified that ttw is caused by a nonsense mutation of a gene encoding nucleotide pyrophosphate (NPPS). NPPS generates Pi and pyrophosphate [34]. Thus, the dysfunction of NPPS might result in phosphate metabolism. Hypophostemic rickets is characterized by a low serum phosphate level. Patients with hypophosphatemic rickets/osteomalacia and hypothyroidism frequently develop ectopic ossification in the spinal ligament as well as in the tissue around the tendon [40,41]. It has been reported that siblings with untreated familial hypophosphatemic vitamin D-resistant rickets develop OPLL [42]. Dysfunction of NPPS might cause ectopic bone formation by disrupting the Pi metabolism. In the current study, a negative correlation was found between the serum concentration of Pi and the severity of OPLL, as defined by the OS index. This might indicate that severe ossified lesions in the spinal canal develop in patients with OPLL, when the patient’s serum concentration of Pi is low. However, the relationship between the serum concentration of Pi and the severity of OPLL is controversial. It remains to be clarified which is the cause and which is the result. Further study is necessary to resolve this issue.

Although the serum concentration of hs-CRP in the progression group was higher than that in the non-progression group, it is still controversial which is cause or result. This means that there is no answer as to whether the local inflammation causes OPLL progression or it is the OPLL progression which results in the elevation of hs-CRP. If local inflammation is a key factor in OPLL pathology, it might be possible to prevent OPLL occurrence and progression by using anti-inflammatory agents. The findings of a systematic review and meta-analysis suggested that non-steroidal anti-inflammatory drugs (NSAIDs) might reduce the incidence of heterotopic ossification (HO) after total hip arthroplasty (THA) [43]. The review also pointed out that NSAIDs might increase the risk of gastrointestinal side effects. We also are wary of NSAIDs-induced renal dysfunction. The selective COX-2 inhibitor, celecoxib, has been proven to be effective for preventing HO after THA [44]. Celecoxib also seems to be effective in preventing HO recurrence after open arthrolysis for post-traumatic elbow stiffness in adults [45]. Indomethacin may be beneficial for decreasing HO volume without increasing the incidence of nonunion in patients with acetabular fracture [46]. As for the use of NSAIDs for spinal pathology, one study reported that there was a low rate of HO in the NSAIDs group compared to the non-NSAIDs group after cervical arthroplasty, although it did not reach statistical significance [47]. Locally delivered indomethacin [48] and Imatinib [49], which is an inhibitor of platelet-derived growth factor, have been tried to prevent HO in animal studies. Thus, it might be reasonable to use NSAIDs for the prevention of OPLL. Further, considering the clinical trials for cardiovascular diseases, several anti-inflammatory medications have been tried for the prevention of the cardiovascular risk based on the previous analysis of hs-CRP. Low-dose aspirin has been used for the prevention of cardiovascular disease, despite that it has been reported that aspirin is not effective [50]. Low-dose methotrexate has been used for the prevention of coronary artery disease [51]. Various statins and Canakinumab, human monoclonal antibody against interleukin 1β, have been used in attempt to prevent atherosclerotic disease [52,53]. Regarding the prevention of heterotopic ossification following total hip arthroplasty, it was recently reported that naproxen is effective in reducing the prevalence of heterotopic ossification [54]. Thus, anti-inflammatory medications might be effective for the prevention of OPLL. Another treatment might be to supply phosphorus. This idea might be considered due to the fact that the patients with OPLL have a low concentration of Pi compared to the controls. However, phosphorus is contained in many daily foods [55]. The Framingham Offspring study demonstrated that there is a risk of low BMD in adult women due to the intake of phosphorus from cola [56]. Thus, phosphorus intake from the diet might not be recommended. 1,25(OH)_2_D promotes Pi reabsorption in the kidney and FGF-23 signals regulate kidney Pi reabsorption and 1,25(OH)_2_D production. The development of preventive measures based on the analysis of 1,25(OH)_2_D and FGF-23 signals might be considered in future studies.

There are several limitations in this study. 1) The serum concentration of the biomarkers analyzed checked at just one point. We took the patient’s blood sample at the visit to our university hospital. Evaluation at several time points might be needed. A longitudinal study is necessary to confirm the stability of the data. 2) It is not evident whether local inflammation occurs at the site of OPLL or not. It might be possible to measure the inflammatory cytokines, such as TNF-α and IL-6 from the tissues surrounding ossified lesions of OPLL. 3) Regarding the meaning of the fact that the hs-CRP was increased and Pi decreased in the OPLL group and the hs-CRP was increased in the OPLL progression group, it remains controversial whether these findings reflect the cause or result, as described above. 4) We could not evaluate the specificity of hs-CRP regarding the pathogenesis of OPLL. These are issues for the future.

In conclusion, the fact that hs-CRP in the patients with OPLL was higher compared to the controls, and hs-CRP in the OPLL progression group was higher than in the non-progression group, indicating that inflammation might occur in OPLL. The serum concentration of Pi was lower in the patients with OPLL and a negative correlation was found between the serum concentration of Pi and the severity of OPLL. We believe that these findings represent new knowledge which will facilitate the understanding of the local pathology of OPLL.

## Supporting information

S1 File Data of comparison between OPLL case and control. (XLSX)

S2 File Data of the comparison between OPLL progression group and non-progression group. (XLSX)
